# Endovascular treatment of acute gastrointestinal bleeding from a large splenic artery pseudoaneurysm: case report and literature review

**DOI:** 10.1590/1677-5449.005517

**Published:** 2018

**Authors:** Paulo Roberto Prette, Felipe Borges Fagundes, Livia Ramos Carvalho Marchon, Rodrigo de Rezende Teixeira Maciel, Igor Miguel Martins, Cristina Ribeiro Riguetti-Pinto

**Affiliations:** 1 Universidade do Estado do Rio de Janeiro – UERJ, Hospital Universitário Pedro Ernesto – HUPE, Departamento de Cirurgia Vascular e Endovascular, Rio de Janeiro, RJ, Brasil.; 2 Curso de Formação em Cirurgia Endovascular – Endocurso, Rio de Janeiro, RJ, Brasil.; 3 Vascularis Centro de Angiologia e Cirurgia Endovascular, Rio de Janeiro, RJ, Brasil.

**Keywords:** endovascular surgery, pseudoaneurysm, splenic artery, therapeutic embolization, pancreatitis, aneurysm

## Abstract

Pseudoaneurysm of the splenic artery is a rare entity, with little more than 150 cases described in the literature. Pancreatitis is the most common etiology, followed by trauma. In contrast with true aneurysms, pseudoaneurysms are frequently symptomatic, with a 47% risk of rupture and 90% mortality if left untreated. We describe the case of a 48-year-old female patient who suffered a gastrointestinal hemorrhage associated with acute-on-chronic pancreatitis. During workup, endoscopy revealed signs of recent bleeding and magnetic resonance angiography of the abdomen showed a large pseudoaneurysm of the splenic artery. The patient underwent endovascular treatment with microcoil embolization and no further bleeding episodes occurred. Endovascular treatment is now an effective option with low morbidity and mortality and success rates in the range of 79-100%, making it a viable technique for patients with active abdominal inflammation. We conducted a review of endovascular techniques and embolization agents used to treat this pathology.

## INTRODUCTION

 True aneurysms involving the visceral arteries are uncommon, with incidence below 0.8%, 1 ^-^ 3 and pseudoaneurysms are even rarer. In both cases, the splenic artery (SA) is the most common site. 4 ^-^ 6 Other sites of visceral pseudoaneurysms, in order of frequency, are the hepatic artery and the celiac artery, among others. 4

 A pseudoaneurysm cannot be classified as a true aneurysm because it is not surrounded by all three layers of the artery wall – intimal, media, and adventitial. 3 The causes of pseudoaneurysms are generally secondary, such as trauma, local infection, and inflammatory pathologies, 7 ^-^ 10 in addition to idiopathic causes. 7 The absence of at least one of the arterial layers means that pseudoaneurysms involve a higher risk of rupture than true aneurysms. 10 ^,^ 11

 A pseudoaneurysm of the splenic artery (PASA) is generally symptomatic. Digestive hemorrhage is the most common symptom 2 and is often associated with hemodynamic instability. 4 In the past, ligature of the SA and splenectomy combined or not with partial pancreatectomy were the most often employed treatments. However, newer, less invasive methods are preferred because they are associated with better results and lower morbidity 11 and are widely accepted as first line treatment. 2 We describe the case of a patient with hematemesis and hemodynamic instability, diagnosed with a large pseudoaneurysm of the SA secondary to acute-on-chronic pancreatitis. We present the technique employed for minimally invasive treatment and discuss the different treatment options. 

## CASE REPORT

 A 48-year-old female patient with chronic alcoholic pancreatitis was admitted via the emergency department with a history of hematemesis. Initial tests revealed significantly elevated pancreatic enzymes, compatible with acute exacerbation of chronic pancreatitis. She underwent elective upper digestive endoscopy (UDE), which reveled a gastric swelling suggestive of extrinsic compression. The upper digestive hemorrhage recurred, causing hemodynamic instability. Initial volume resuscitation measures were successful and an urgent UDE showed the swelling covered with mucosa indicative of infiltrate, an oval-shaped erosion with a hematin background located on the large curvature of the distal stomach, and a large clot occupying the entire gastric fundus, with no signs of active bleeding. 

**Figure 1:**
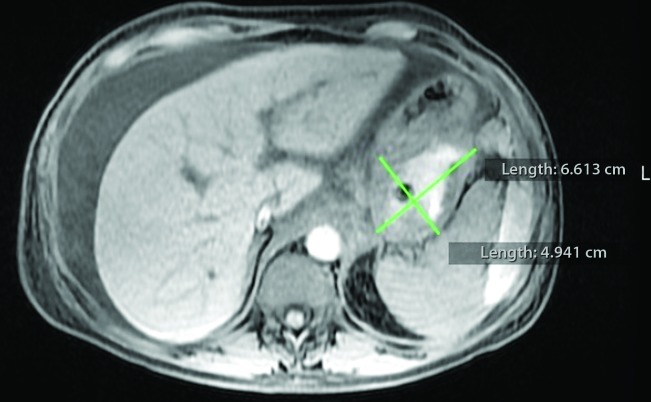
Magnetic resonance image of the abdomen in T1 with fat saturation after injection of contrast showing a large pseudoaneurysm of the splenic artery measuring 6.6 x 4.9 cm in contact with the posterior wall of the stomach.

**Figure 2:**
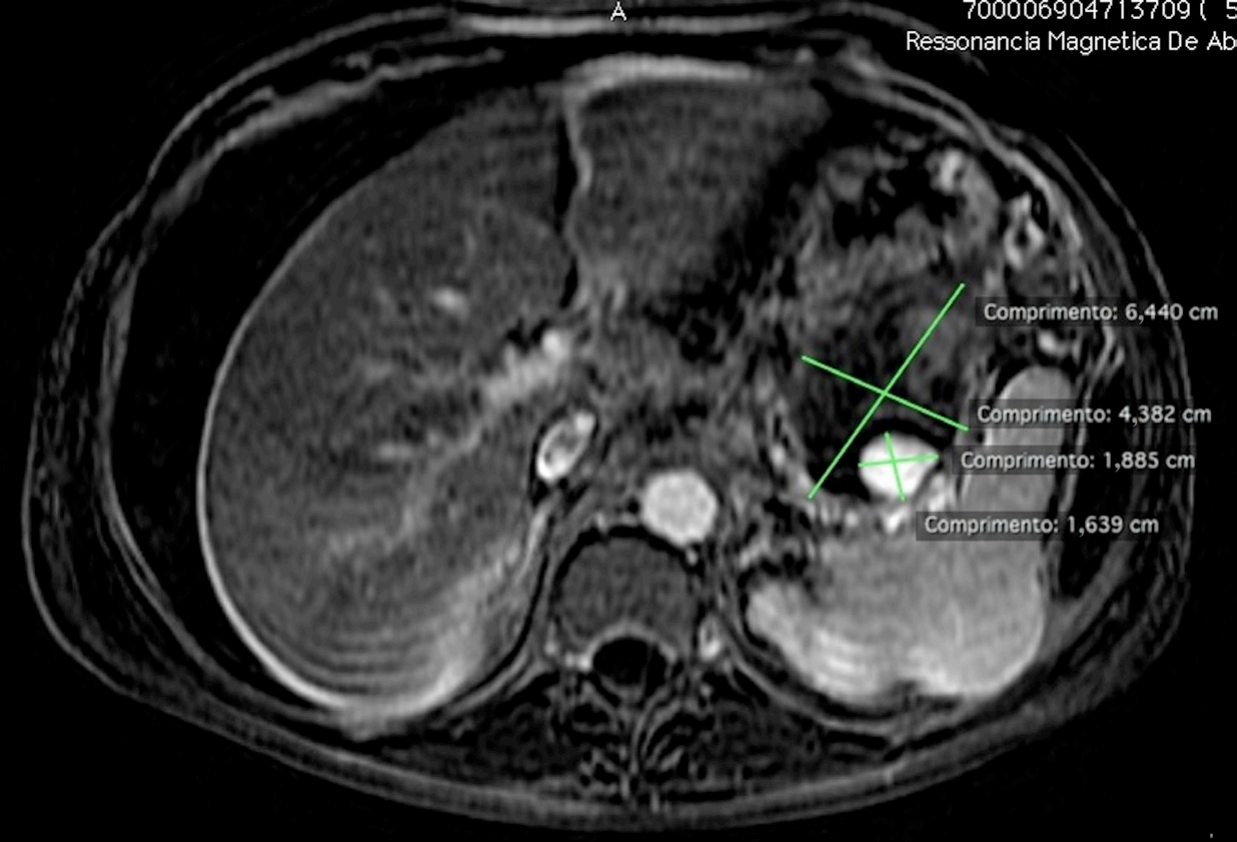
Magnetic resonance image of the abdomen in T1 with fat saturation after subtraction showing a pseudoaneurysm of the splenic artery close to the splenic hilum, surrounded by an oval-shaped mass with thick/hematic content.

 Magnetic resonance imaging (MRI) revealed a saccular aneurysmal dilatation of the SA measuring around 2.0 x 1.6 cm. It was surrounded by an oval-shaped mass with thick/hematic content, suggesting a pseudoaneurysm of around 6.4 x 4.3 cm, in contact with the posterolateral wall of the gastric body ( Figures 1 and 2 ). 

**Figure 3:**
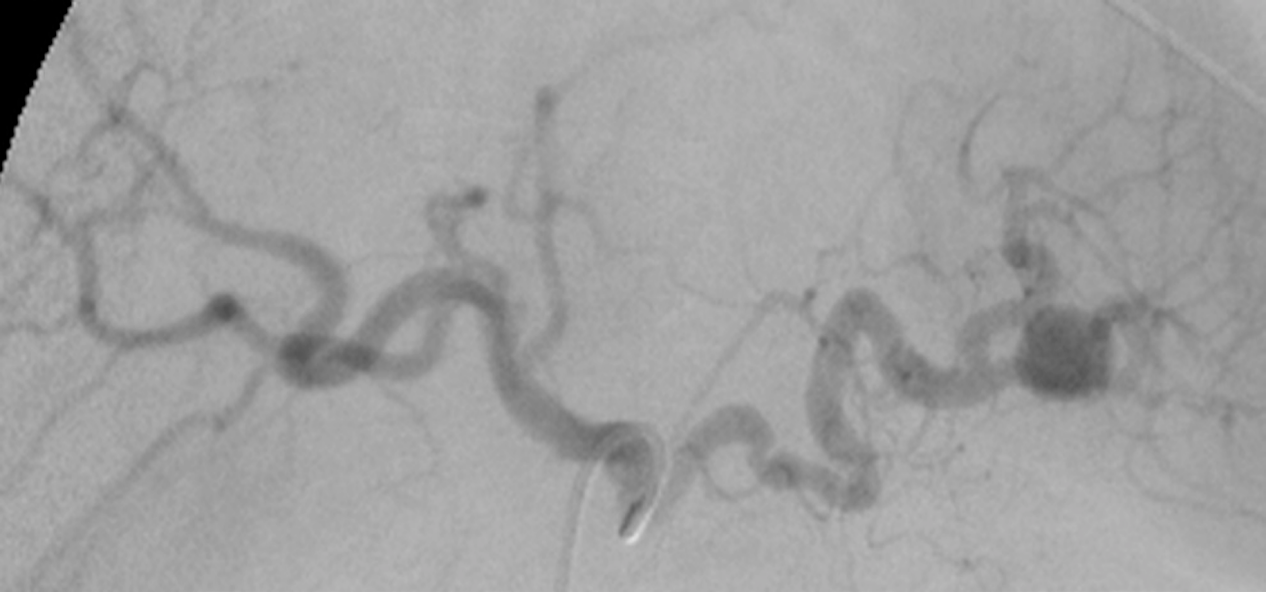
Digital subtraction angiography by selective catheterization of the celiac trunk in PA showing the pseudoaneurysm in the inferior branch of the splenic artery and intense tortuosity of the splenic artery.

**Figure 4:**
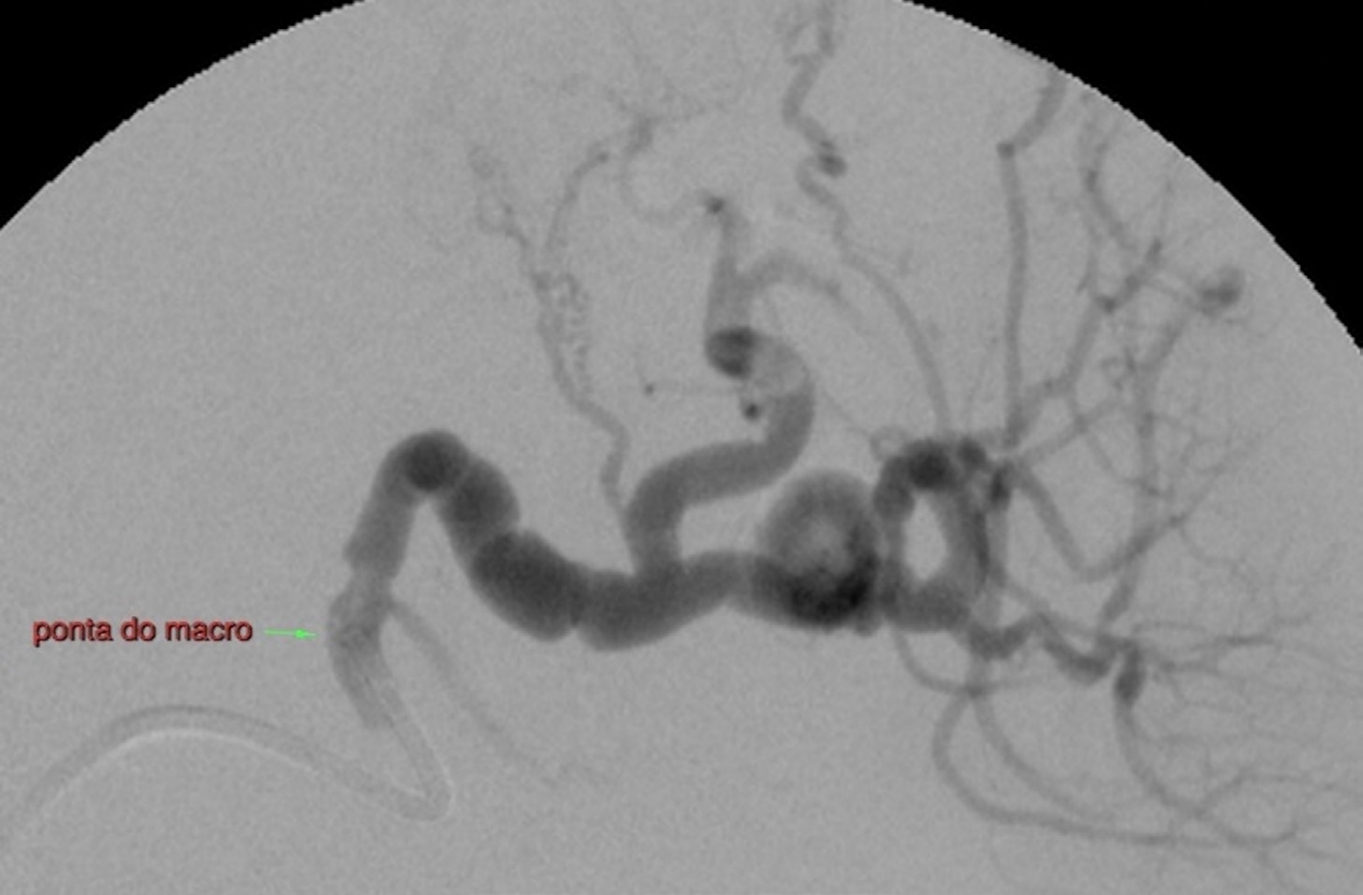
Digital subtraction angiography by superselective catheterization of the splenic artery with a Simmons 1 catheter confirming the topography of the pseudoaneurysm and determining the working angle.

**Figure 5:**
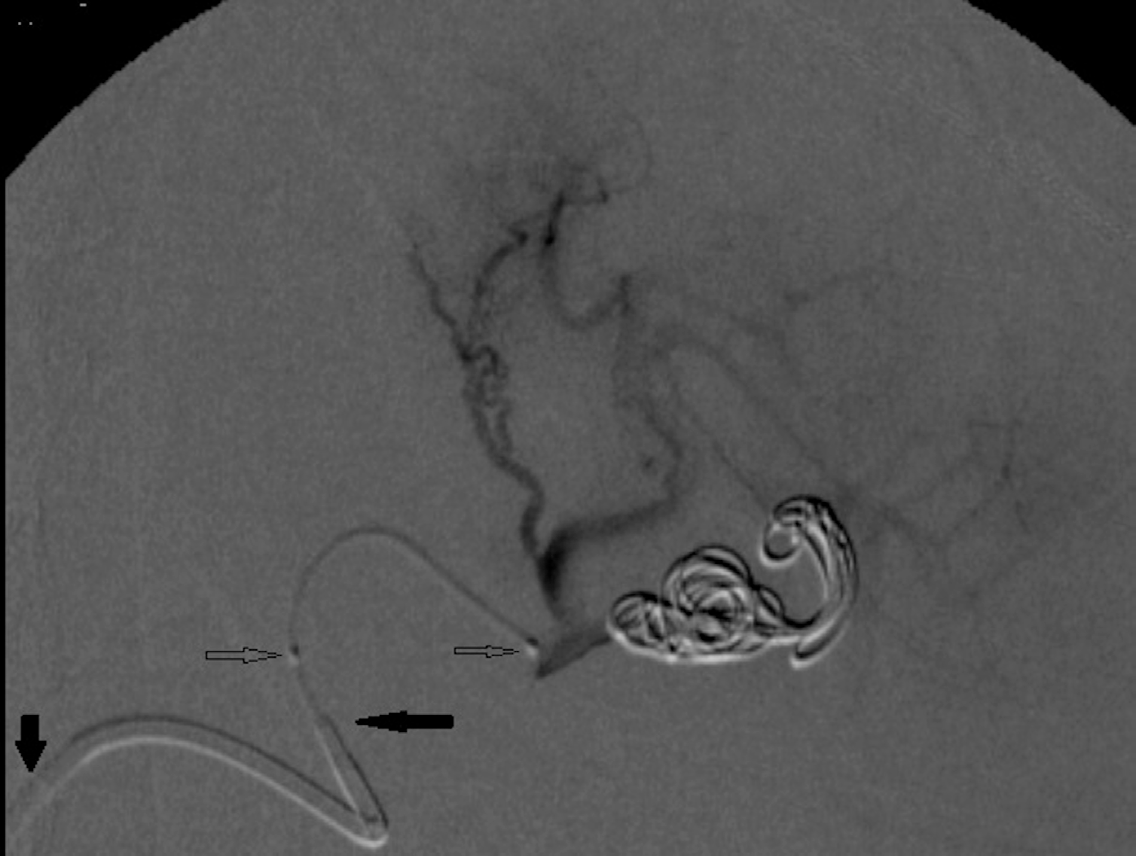
Digital subtraction angiography by microcatheterization of the distal splenic artery showing successful embolization with microcoils positioned in the neck distal of the pseudoaneurysm (one 8 x 30 mm unit) and in the interior and proximal to the pseudoaneurysm (two 6 x 30 mm units). Patency of the superior branch of the splenic artery and short gastric arteries, maintaining arterial supply to the spleen, can be observed. The tips of the two macros (vertical shaded arrow: guide catheter; horizontal shaded arrow: Simmons 1) and the two distal markings on the microcatheter (horizontal unshaded arrows) can be observed.

**Figure 6:**
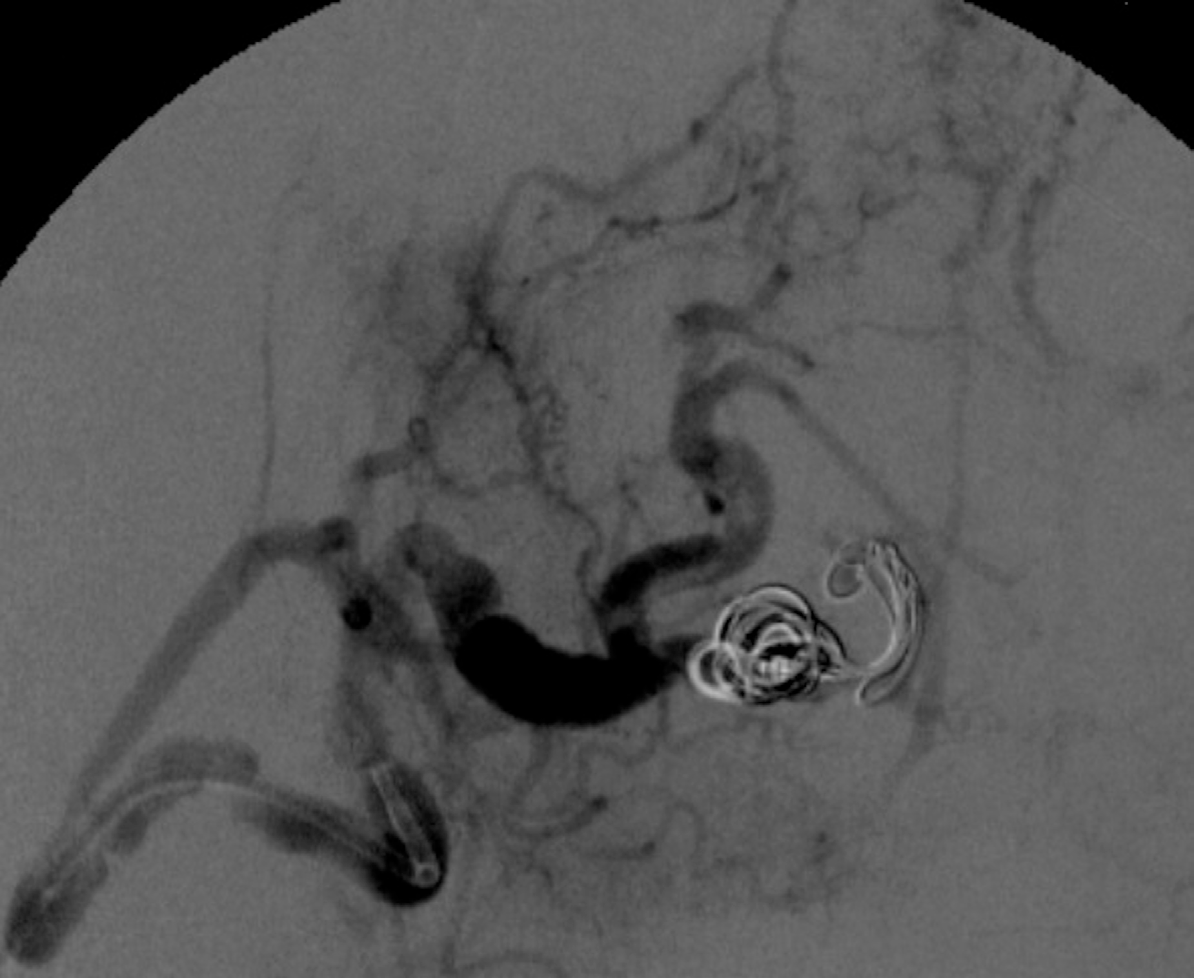
Final control digital subtraction angiography showing exclusion of the pseudoaneurysm and absence of contrast leakage, with patency of the superior and collateral branches.

 Having been diagnosed with PASA, the patient was treated with percutaneous embolization via the right common femoral artery. The SA was accessed using a coaxial technique with a guide catheter over a Simmons 1 (SIM 1) angiographic catheter over a 0.035” x 260 cm hydrophilic guidewire ( Figures 3 and 4 ). The decision was taken to embolize using controlled release microcoils via microcatheter, one 8 x 30 mm unit distal of the neck and two 6 x 30 mm units proximal to the neck ( Figure 5 ). Control angiography showed total occlusion of the pseudoaneurysm ( Figure 6 ). 

 The patient remained hemodynamically stable and there were no complications related to the embolization technique, except for mild pain in the left hypochondrium on the first day after the operation. She remained hemodynamically stable for the next 2 weeks, with no further episodes of bleeding. However, because of her severe clinical status, she died from pulmonary sepsis. 

## DISCUSSION

 Although PASA is the most common type of visceral aneurysm, it is still a rare entity. 1 ^,^ 11 If it is not treated promptly, the risk of rupture is in the range of 37-47% 12 ^,^ 13 and the mortality rate is 90%. 5 ^,^ 12 ^,^ 13 In the case described here, the patient was admitted because of symptoms related to PASA rupture: upper digestive hemorrhage with hemodynamic instability. 

 In a large series from the Mayo Clinic that was published in 2003, Tessier et al. 4 reported ten PASA cases over 18 years, recommending treatment in all cases, because they found no relationship between size and risk of rupture. In the literature from Brazil, we found two reports of PASA cases and two cases of gastroduodenal pseudoaneurysm, all progressing to bleeding secondary to rupture. 13 ^-^ 16

 The most common causes of PASA include local inflammatory states (primarily chronic and acute pancreatitis) and trauma. 4 ^,^ 5 ^,^ 11 ^-^ 13 ^,^ 17 Tessier et al. 4 reported associations with pseudocysts in 41% of cases of pancreatic PASA. Among other less frequent causes, Hartman et al. 17 described a PASA associated with scleroderma and gastric ulcer, with no history of pancreatitis. They assumed that the macrovascular disease of the scleroderma reduced the elasticity of vessels and caused microangiopathy of the *vasa vasorum*, leading to ischemia of the vascular wall, predisposing to pseudoaneurysm formation. Schatz et al. 11 describe rupture of an idiopathic PASA, progressing to hemorrhagic shock. In the present report, the condition was associated with acute-on-chronic pancreatitis and a history of chronic alcoholism. 

 It is believed that the pathophysiology of PASA can be explained in three ways: in cases of pancreatic or peripancreatic inflammation, the wall of the SA is thought to undergo digestion by pancreatic enzymes, with consequent weakening of the artery wall 4 ^,^ 18 ^,^ 19 ; in trauma, the second most common cause, the rapid deceleration would result in damage to the intima and the elastic layer of the SA, predisposing it to formation of a pseudoaneurysm 4 ^,^ 10 ^,^ 18 ; and in cases related to pancreatic pseudocysts, these may erode the artery wall and cause a fistula from the artery to the mucosa of the gastrointestinal tract or the interior of the pseudocyst. 12 ^,^ 18

 In chronic pancreatitis, in addition to occurrence of PASA, the most common form (40%), other arteries may also be involved, such as the gastroduodenal artery (30%); the pancreaticoduodenal artery (20%); the left gastric artery (5%); and the common hepatic artery (2%). 18 In an article containing case reports and a review of the literature, Tessier et al. 4 list the most common symptoms as gastrointestinal hemorrhage and abdominal pains. Other symptoms include nausea, vomiting, lumbar pain, chest pain, and abdominal masses. 

 Since the most prevalent clinical presentation of this pathology is gastrointestinal bleeding, the first examinations ordered are generally diagnostic endoscopy and colonoscopy. Splenic artery pseudoaneurysms may rupture into the interior of a pseudocyst, into the peritoneal cavity, or into the retroperitoneal space, without digestive hemorrhage, or may form a fistula into the intestinal lumen, 20 the bile duct (hemobilia), or the interior of the pancreatic duct, 13 ^,^ 18 a condition known as *hemosuccus pancreaticus*. 16 ^,^ 21 ^-^ 23 In the case described here, a UDE was conducted to investigate hematemesis, bleeding directly into the gastrointestinal tract. Endoscopic treatment was not performed at that juncture because there was no evidence of active bleeding. 

 To arrive at a conclusive diagnosis, an MRI was conducted, showing a large PASA. In this situation, the diagnostic options include many different imaging exams, of which computed tomography angiography (angioCT) and MRI are the best noninvasive examinations currently available. 8 ^,^ 19 While MRI has the advantage of not requiring iodinated contrast and enables an equally effective study as angioCT, 8 its limitations include contraindications for patients who have pacemaker or metallic aneurysm clips, those with claustrophobia, and people who are unable to hold their breath. 19 On computed tomography (CT) without contrast, a PASA can be identified as a focal region of intensification surrounded by hypodense fluid. The PASA will have increased attenuation without contrast, but its perfused portion is strongly intensified with contrast. 19 With multi-detector CT, the ability to perform multiplane reconstructions with submillimetric slice thicknesses, allied with acquisition of angiotomographic images, not only enables us to confirm diagnosis, but also to infer which vessel is involved and to define the surgical strategy. 8 ^,^ 21 In the case described here, initial diagnosis and planning of surgery were accomplished using MRI, a diagnostic method that was available at the time of clinical presentation. 

 Color Doppler ultrasonography is generally the first imaging exam used for diagnosis of peripheral pseudoaneurysms, 8 since it is a noninvasive examination that offers the benefits of wide availability, low cost, and real-time assessment, without using contrast. 4 However, this examination is operator-dependent and can suffer interference from obesity and acoustic shadows, caused by intestinal gas or atherosclerosis. 19 Additionally, small lesions may go unnoticed. 4 ^,^ 19 In the case described here, MRI was ordered immediately, because there was a high level of suspicion of PASA and the case was an emergency, demanding a highly specific diagnostic method. 

 Once in possession of the greatest quantity of information possible about the anatomy and grade of the pathology, in conjunction with an analysis of the clinical conditions of the patient, it is possible to choose the best treatment with confidence. In the past, ligature of the SA, with or without revascularization, and splenectomy combined or not with partial pancreatectomy were the usual treatments. 9 ^,^ 11 ^,^ 24 However, surgical treatment is associated with high mortality rates (5-25%), 10 and there are cases with relative contraindications against laparotomy, such as those involving acute pancreatitis. Distal pancreatectomy, with or without splenectomy, has a mortality rate of 10-50%, 1-year survival of 100%, and 5-year survival of 85%. 18 Tessier et al. 4 advise that pseudoaneurysms associated with formation of pancreatic pseudocysts are best treated by surgical excision because of the difficulty of embolization of the large pseudocyst cavity. For patients in whom the pseudoaneurysm ruptures into the free cavity of the pseudocyst, embolization may be sufficient to address hemodynamic instability, enabling definitive surgical treatment later. The main indications for invasive surgical treatment are patient instability precluding the angiographic procedure, inability to conduct embolization, and persistent or recurrent bleeding. 6 In the case described here, the patient’s anatomy was favorable for endovascular treatment and she had acute-on-chronic pancreatitis, an inflammatory condition that made an open surgery approach more difficult. 

 The new less invasive methods are preferable, with better results and lower morbidity. 9 ^,^ 10 ^,^ 11 ^,^ 25 ^,^ 26 The embolization technique is associated with a reduced need of transfusion and shorter hospital stay, when compared with open surgery. 24 ^,^ 27 Batagini et al. 27 conducted a retrospective study of patients with aneurysms and pseudoaneurysms of visceral arteries who were treated surgically between January 2007 and April 2015 by the vascular surgery department at the Cleveland Clinic, finding evidence that minimally invasive treatments resulted in shorter operation duration, reduced blood loss transfusion rate and shorter length of hospital stay when compared with conventional surgical treatment. They did not find differences in rates of intraoperative or postoperative complications such as parenchymal ischemia, acute myocardial infarction, acute renal failure, deep venous thrombosis, or respiratory complications. There was also no significant difference in rates of technical success of the different types of procedure. There was no difference in the clinical success rates of the two approaches during mid-term follow-up – a mean of 16 months. In their conclusions, the authors emphasized the need for long-term follow-up studies to evaluate the durability of endovascular treatment, but stated that the successful initial results and low reintervention rates showed that the minimally invasive approach was safe and feasible. It should be considered that the advantages of a percutaneous approach may be much more obvious in cases of hostile abdomen, such as in the patient described here. 

 Percutaneous arteriography is considered the gold-standard method and the most sensitive examination for identifying aneurysms and pseudoaneurysms. 6 It enables a detailed vascular assessment and is recommended before any elective surgical procedure, since it confirms diagnosis and determines the site of involvement with sensitivity of 94-100%. 6 ^,^ 24 The embolization technique is safe with low invasivity, but it has a significant failure rate, with 5% reperfusion after treatment. 7 Spiliopoulos et al. analyzed the results of endovascular treatment of visceral aneurysms and pseudoaneurysms at three European interventional radiology departments from 2000 to 2010. 3 Overall, 21 visceral aneurysms and 37 visceral pseudoaneurysms were treated. They reported technical success in 100% of cases, with a 6.1% reintervention rate in the pseudoaneurysm group and 14.2% in the true aneurysm group. The overall mortality rate for the procedures was 3% in the pseudoaneurysm group and 0% in the aneurysm group. They concluded that endovascular treatment is safe and effective, with low morbidity and mortality. 

 Endovascular treatment can be accomplished using a range of techniques, such as coils, n-butyl cyanoacrylate (NBCA), ethylene-vinyl alcohol copolymer (Onyx®), thrombin, or covered stents. 8 Since pseudoaneurysms involve a loss of integrity of the vascular wall, the embolization itself should not be chosen as the only treatment, since the emboligenic content will be contained within a virtual wall and there is a serious risk of repeat rupture. There is a report in the literature of migration of a covered stent into the interior of the stomach after treatment of a splenic pseudoaneurysm. 28 The percutaneous procedure for embolization consists of superselective catheterization of the artery involved and distal and proximal embolization of the lesion and the endoluminal pseudoaneurysm sac, using coils or NBCA. 6 It is essential to exclude both the afferent and the efferent vessels to reduce the risk of anterograde and retrograde reperfusion. 3 ^,^ 7 ^,^ 26 The need to position the coils in the efferent branch and then the afferent branch to achieve complete exclusion means that perfusion of the organ will be at least partially maintained by collateral vessels. 26

 Coils and microcoils are the agents of choice for treatment of aneurysms and pseudoaneurysms, 3 ^,^ 25 ^,^ 29 but their use can result in incomplete or ineffective embolization because of the following causes: (1) pseudoaneurysm fed by narrow or tortuous arteries; (2) collateral network supplying the pseudoaneurysm; (3) inadequate coil packing or “nesting” of coils; or (4) coagulation deficiency. 3 ^,^ 25 Two endovascular techniques using coils are recommended. The coil packing technique is used to embolize the aneurysm sac itself. The coil trapping exclusion technique consists of occlusion of proximal and distal arteries to prevent refilling. 7 ^,^ 8

 Kingma et al. 7 describe a case in which the exclusion technique was used but, because of intense vasospasm consequent to progression of the catheter tip, embolization of the afferent artery was not possible. The same problem prevented placement of a covered stent, and open surgical treatment was needed with splenectomy and removal of the tail of the pancreas. The authors suggest that using a microcatheter could have enabled distal catheterization without vasospasm or dissection of the artery. In the present case, we used a coaxial technique to position the working system in the proximal segment of the SA and the microcatheterization technique with a microcatheter and microguidewire for embolization of the artery afferent and efferent to the pseudoaneurysm ( Figure 5 ). 

 N-butyl cyanoacrylate is a clear liquid at room temperature that solidifies rapidly in contact with ionic fluids such as saline solution and blood. 29 It offers two advantages over coils as an emboligenic agent: immediate occlusion of the artery embolized and the possibility of more distal placement than can be achieved with microcoils. 9 Other benefits of the glue include: low viscosity, enabling distal embolization when catheterization is impossible because of anatomic difficulties; embolization of collateral arteries; and no dependence on coagulation activity. 25 However, NBCA may polymerize too early and cause the catheter to adhere to the vessel. Late polymerization may also occur, resulting in irreversible distal emboli. 30 Thus, effective use of NBCA is dependent on control over the volume injected and the velocity of infusion, 9 making execution difficult for those who are unfamiliar with the method. In the present case, with a high arterial flow in a medium caliber vessel, use of liquid glue with high polymerization was contraindicated by the high risk of distal emboli and consequent splenic ischemia. 

 Onyx® is another alternative to coil embolization that can now be used in a similar manner to NBCA. There are reports in the literature of indications for neurointerventions to directly embolize the aneurysm sac. However, the high density formula (500) used for this purpose was withdrawn from the market. The presentations currently available in Brazil are of lower densities, for peripheral use. Onyx® is a non-adhesive liquid embolic agent dissolved in dimethyl sulfoxide (DMSO). 30 When the DMSO diffuses out of the mixture, Onyx® becomes a spongy, elastic solid, causing exclusion of the sac or vessel by filling it with an elastic polymer which molds to the wall, occupying the entire lumen. There is no risk of permanent adhesion of the catheter to the wall, which is an inherent risk when using NBCA. Onyx® has been used successfully in aneurysms that do not meet the indications for coils or when embolization with other materials fails. However, to date there are few long-term data on indications for true or pseudo visceral aneurysms. 30

 Success rates of embolization reported in the literature are in the range of 75-100% with morbidity estimated at 14-25%. 26 Ballinas-Oseguera et al. 6 state that in 93% of cases of successful embolization, hemorrhage is controlled at the first arteriography and in 100% after a second session. These data are similar to those from a study by Laganà et al., 26 who treated 25 out of 29 patients with aneurysms of visceral arteries by embolization and achieved immediate exclusion of the aneurysms in all cases. Two of the four remaining patients were treated with covered stents and the other two by injection of thrombin and embolization of the afferent artery with coils. The rate of complications was 27.6% (seven cases of splenic ischemia and one occlude covered stent), and in 10.3% of cases reperfusion occurred within the first month of follow-up, all treated successfully with endovascular techniques. The primary success rate was 89.7% and the secondary success rate was 100%. The reperfusion rate was 5% and all cases occurred soon after treatment. No cases of recanalization were reported in the medium or long term. 

 Disadvantages of embolization of a PASA include a 40% rate of splenic infarction. Major splenic infarctions are more common after embolization of the distal third of the splenic artery or hilum. These patients may complain of intense abdominal pains and require admission to hospital for pain control. They may need splenectomy or drainage of splenic abscesses. 4 In the case described here, the pseudoaneurysm was located in the lower branch of the SA, and there were no signs of infarction of the organ, probably because of the preserved perfusion via the superior branch. There are reports of migration of materials used in embolization to the visceral arteries, aorta, and gastrointestinal tract, and exclusion of the distal circulation can compromise the organ’s function. 10 Extra-arterial migration of emboligenic content occurs if embolization of the aneurysm sac is performed, since the pseudoaneurysm does not have an artery wall. This is why we recommend using the coil trapping technique for afferent and efferent arterial exclusion, as described above. 

 Exclusion of aneurysms with covered stents offers alternatives to the disadvantages of embolization, because it enables effective hemostasis and thrombosis of the pseudoaneurysm without risk of rebleeding, with maintenance of the lumen and vascular flow to the organ. 2 ^,^ 26 ^,^ 30 It is particularly indicated in cases of pseudoaneurysms with wide necks. 8 The technique offers reduced morbidity when compared with open surgery and embolization. However, its use is restricted to appropriate anatomies. Favorable anatomic conditions include minimum distal and proximal neck size (5-15 mm de length), adequate caliber, 2 proximal location of the SA lesion, and absence of arterial branching. 10 The intense tortuosity of the splenic artery, the high incidence of this type of lesion close to the hilum, and the rigidity of the delivery devices mean that the anatomic conditions are frequently not ideal. 3 ^,^ 26 ^,^ 30 Treatment with a covered stent is generally difficult because of the small diameters of the visceral arteries. Causes of failure reported include recanalization by collaterals and vascular remodeling. 26 Intimal hyperplasia may also occur at the edges of the covered stent. 30 Sepsis is not an absolute contraindication against their use, but does demand surveillance during postoperative follow-up. 8 ^,^ 10 Reed et al. conducted a retrospective study of patients with aneurysms of the splenic artery treated between 2009 and 2014 with covered stents or salvage embolization after failure of the first method. The series comprised 10 patients, with mean aneurysm diameter of 2.8 ± 1.3 cm, mean size of covered stent of 6x100 mm, and a mean of 1.5 stents implanted (range: 1-4). The technical success rate was 80% and the two technical failures were related to the intense tortuosity of the vessels. In those two cases, treatment was achieved with coils or Amplatzer plugs. In the procedure described here, we chose the embolization with microcoils technique because of the intense tortuosity of the SA, using the coaxial technique to increase the support provided to the working systems. 

 Shrivastava et al. 31 reported a case treated by direct ultrasound-guided percutaneous puncture of the pseudoaneurysm. Intense tortuosity of the SA prevented selective catheterization. Embolization was achieved using coils, with technical success and maintenance of SA patency. There are also reports of successful ultrasound-guided thrombin injection into visceral pseudoaneurysms. 8

 Irrespective of the technique used, the great majority of the complications reported in the literature comprise ischemia of the upper part of the gastrointestinal tract, in 30-66% of cases. Splenic infarction can occur and may resolve spontaneously or may require splenectomy; 2 ^,^ 18 technical complications such as migration of embolizing materials or covered stents; 28 or complications affecting the surgical access or puncture site. Patients may have pain, fever, and transitory elevation of pancreatic or hepatic enzymes (post-embolization syndrome). 26 In the present case, the patient maintained hemodynamic stability, without complications related to the embolization technique, except for mild pain in the left hypochondrium on the first day after the operation. 

## CONCLUSIONS

 Minimally invasive treatment is described as effective for treatment of PASA, and other visceral pseudoaneurysms, including in cases with hemodynamic instability. 3 The choice of technique should be based on the patient’s clinical and anatomic conditions and on the surgeon’s or interventional radiologist’s skills and familiarity with the method. It should be stressed that it is important to treat not only the pseudoaneurysm itself, but also the afferent and efferent vessels to reduce the chances of recurrence. 
