# Supplementary material for: Endovascular treatment of acute gastrointestinal bleeding from a large splenic artery pseudoaneurysm: case report and literature review
Source: J Vasc Bras. 2018 Jul-Sep;17(3):234–42. doi: 10.1590/1677-5449.005517 (PMC6326128; doi:10.1590/1677-5449.005517)
Supplement: Translation

# Translation: Tratamento endovascular da hemorragia digestiva aguda por volumoso pseudoaneurisma esplênico: relato de caso e revisão da literatura

**Authors:** Paulo Roberto Prette, Felipe Borges Fagundes, Livia Ramos Carvalho Marchon, Rodrigo de Rezende Teixeira Maciel, Igor Miguel Martins, Cristina Ribeiro Riguetti-Pinto

**Affiliations:** 1 Universidade do Estado do Rio de Janeiro – UERJ, Hospital Universitário Pedro Ernesto – HUPE, Departamento de Cirurgia Vascular e Endovascular, Rio de Janeiro, RJ, Brasil.; 2 Curso de Formação em Cirurgia Endovascular – Endocurso, Rio de Janeiro, RJ, Brasil.; 3 Vascularis Centro de Angiologia e Cirurgia Endovascular, Rio de Janeiro, RJ, Brasil.

**Keywords:** cirurgia endovascular, pseudoaneurisma, artéria esplênica, embolização terapêutica, pancreatite, aneurisma

## Abstract

O pseudoaneurisma da artéria esplênica é uma entidade rara, com pouco mais de 150 casos descritos na literatura. A pancreatite é a etiologia mais comum, seguida do trauma. Em contraposição ao aneurisma verdadeiro, esse pseudoaneurisma é frequentemente sintomático, com risco de ruptura de 47% e mortalidade de 90%, quando não tratado. Descrevemos o caso de uma paciente de 48 anos que apresentou hemorragia gastrointestinal associada a pancreatite crônica agudizada. Durante investigação, a endoscopia evidenciou sinais de sangramento recente, e a angiorressonância de abdome observou volumoso pseudoaneurisma da artéria esplênica. Foi submetida a tratamento endovascular com embolização com micromolas, não apresentando novos episódios de sangramento. Atualmente, o tratamento endovascular é efetivo com baixa morbimortalidade e taxas de sucesso de 79-100%, sendo uma técnica viável para pacientes com processo inflamatório abdominal em franca atividade. Realizamos uma revisão das técnicas endovasculares e agentes embolizantes usados para o tratamento dessa patologia.

## INTRODUÇÃO

 Os aneurismas verdadeiros das artérias viscerais são incomuns, com incidência menor que 0,8% 1 ^-^ 3 , sendo os pseudoaneurismas ainda mais raros. Em ambos, a artéria mais acometida é a esplênica (AE) 4 ^-^ 6 . Outros sítios de pseudoaneurisma visceral, em ordem de frequência, são: artéria hepática, artéria celíaca, entre outras 4 . 

 O pseudoaneurisma não pode ser classificado como um aneurisma verdadeiro por não ser revestido pelas três camadas da parede arterial – íntima, média e adventícia 3 . Geralmente, possui uma causa secundária, como trauma, infecção local e patologias inflamatórias 7 ^-^ 10 , além das causas idiopáticas 7 . Devido à ausência de pelo menos uma camada arterial, o pseudoaneurisma possui maior risco de ruptura que o aneurisma verdadeiro 10 ^,^ 11 . 

 O pseudoaneurisma da artéria esplênica (PSAE) é geralmente sintomático. A hemorragia digestiva é o sintoma mais comum 2 , frequentemente associada à instabilidade hemodinâmica 4 . No passado, a ligadura da AE e a esplenectomia associada ou não a pancreatectomia parcial eram os tratamentos usualmente utilizados. Porém, novos métodos menos invasivos são preferidos por apresentarem melhores resultados e menor morbidade 11 , sendo amplamente aceitos como primeira linha de tratamento 2 . Relatamos o caso de uma paciente com hematêmese e instabilidade hemodinâmica, com diagnóstico de volumoso pseudoaneurisma de AE secundário a pancreatite crônica agudizada. Apresentamos a técnica utilizada de tratamento minimamente invasivo e discutimos as diferentes opções terapêuticas. 

## RELATO DE CASO

 Paciente feminina, 48 anos, portadora de pancreatite crônica de etiologia alcoólica, deu entrada no setor de emergência com história de hematêmese. Após exames iniciais, foi constatada elevação importante das enzimas pancreáticas, compatível com quadro de agudização da pancreatite crônica. Foi submetida a endoscopia digestiva alta (EDA) eletiva, que identificou abaulamento gástrico sugestivo de compressão extrínseca. Evoluiu com novo quadro de hemorragia digestiva alta, cursando com instabilidade hemodinâmica. Após medidas iniciais de reposição volêmica com sucesso, foi evidenciada em EDA de urgência a presença de abaulamento recoberto por mucosa de aspecto infiltrado, erosão ovalar com fundo de hematina localizado na grande curvatura do corpo distal do estômago e grande coágulo ocupando todo o fundo gástrico, sem sinais de sangramento ativo. 

 Foi realizada em sequência uma ressonância magnética (RM), que evidenciou dilatação aneurismática sacular da AE medindo em torno de 2,0 x 1,6 cm. Estava circundada por coleção ovalada de conteúdo espesso/hemático, sugerindo pseudoaneurisma com cerca de 6,4 x 4,3 cm, em contato com a parede posterolateral do corpo gástrico ( Figuras 1 e 2 ). 

**Figura 1:** Imagem de ressonância magnética do abdome em T1 com saturação de gordura após injeção de contraste evidenciando volumoso pseudoaneurisma de artéria esplênica de 6,6 x 4,9 cm em contato com a parede posterior do estômago.

**Figura 2:** Imagem de ressonância magnética de abdome em T1 com saturação de gordura após subtração evidenciando pseudoaneurisma da artéria esplênica próximo ao hilo esplênico circundado por coleção ovalada de conteúdo espesso/hemático.

 Com diagnóstico de PSAE, a paciente foi submetida a embolização percutânea via artéria femoral comum direita. O acesso à AE foi realizado por técnica coaxial com cateter guia sobre cateter angiográfico Simmons 1 (SIM 1) sobre fio guia hidrofílico 0,035” x 260 cm ( Figuras 3 e 4 ). Optou-se por embolização com micromolas de destaque controlado, via microcateter, sendo uma unidade de 8 x 30 mm distal ao colo e duas unidades de 6 x 30 mm proximais ao colo ( Figura 5 ). No estudo angiográfico de controle, evidenciou-se oclusão total do pseudoaneurisma ( Figura 6 ). 

**Figura 3:** Angiografia em subtração digital por cateterismo seletivo do tronco celíaco em PA evidenciando pseudoaneurisma no ramo inferior da artéria esplênica e intensa tortuosidade da artéria esplênica.

**Figura 4:** Angiografia em subtração digital por cateterismo superseletivo da artéria esplênica com cateter Simmons 1 confirmando a topografia do pseudoaneurisma e determinando a incidência de trabalho.

**Figura 5:** Angiografia em subtração digital por microcateterismo da artéria esplênica distal revelando sucesso na embolização com micromolas posicionadas no colo distal ao pseudoaneurisma (uma unidade de 8 x 30 mm) e no interior e proximal ao pseudoaneurisma (duas unidades de 6 x 30 mm cada). Observa-se a perviedade do ramo superior da artéria esplênica e gástricas curtas, permitindo a manutenção do suprimento arterial ao baço. Observa-se a ponta dos dois macros (seta cheia vertical: cateter guia; seta cheia horizontal: Simmons 1) e duas marcações distais do microcateter (setas horizontais vazias).

**Figura 6:** Angiografia em subtração digital de controle final revelando exclusão do pseudoaneurisma e ausência de extravasamento de contraste, com perviedade do ramo superior e colaterais.

 A paciente evoluiu com estabilidade hemodinâmica, sem complicações relativas à técnica de embolização, exceto por dor discreta em hipocôndrio esquerdo no primeiro dia pós-operatório. Houve estabilização hemodinâmica nas duas semanas seguintes ao procedimento, sem novo episódio de sangramento. No entanto, devido ao grave quadro clínico, a paciente foi a óbito por sepse pulmonar. 

## DISCUSSÃO

 Apesar de o PSAE ser o aneurisma visceral mais comum, ainda assim é uma entidade rara 1 ^,^ 11 . Caso não seja tratado precocemente, apresenta risco de ruptura de 37-47% 12 ^,^ 13 e taxa de mortalidade de 90% 5 ^,^ 12 ^,^ 13 . Neste caso, a paciente foi internada pelos sintomas relativos à ruptura do PSAE: hemorragia digestiva alta, cursando com instabilidade hemodinâmica. 

 Em uma grande série da Mayo Clinic publicada em 2003, Tessier et al. 4 reportaram dez PSAE em 18 anos, recomendando o tratamento em todos os casos, pois não conseguiram relacionar o tamanho ao risco de ruptura. Na literatura nacional, foram encontrados dois relatos de casos de PSAE e dois de gastroduodenal, todos evoluindo com sangramento por ruptura 13 ^-^ 16 . 

 Entre as causas do PSAE, as mais comuns são os estados inflamatórios locais (principalmente a pancreatite crônica e aguda) e o trauma 4 ^,^ 5 ^,^ 11 ^-^ 13 ^,^ 17 . Tessier et al. 4 relatam associação com pseudocisto em 41% dos casos de PSAE por pancreatite. Entre outras causas menos frequentes, Hartman et al. 17 descrevem um PSAE associado a esclerodermia e úlcera gástrica, sem história de pancreatite. Esses autores supõem que a doença macrovascular na esclerodermia cause diminuição da elasticidade dos vasos e microangiopatia da *vasa vasorum*, ocasionando isquemia da parede vascular, o que predispõe à formação de pseudoaneurisma. Schatz et al. 11 citam a ruptura de um PSAE de causa idiopática, cursando com choque hemorrágico. Neste relato, a paciente apresentou associação com pancreatite crônica agudizada e história de alcoolismo crônico. 

 Acredita-se que a fisiopatologia do PSAE possa ser explicada de três formas: nos casos de inflamação pancreática ou peripancreática, ocorreria a digestão da parede da AE pelas enzimas pancreáticas, com consequente enfraquecimento da parede arterial 4 ^,^ 18 ^,^ 19 ; no trauma, segunda causa mais comum, a rápida desaceleração resultaria em dano da íntima e da camada elástica da AE, predispondo à formação do pseudoaneurisma 4 ^,^ 10 ^,^ 18 ; e nos casos relacionados a pseudocisto pancreático, este poderia erodir para a parede arterial e originar uma fístula da artéria para a mucosa do trato gastrointestinal ou para o interior do pseudocisto 12 ^,^ 18 . 

 Na pancreatite crônica, além da ocorrência de PSAE – mais comum (40%) –, outras artérias também podem ser acometidas, como artéria gastroduodenal (30%); artéria pancreaticoduodenal (20%); artéria gástrica esquerda (5%); e artéria hepática comum (2%) 18 . Em um artigo de relato de casos e revisão de literatura, Tessier et al. 4 apontam como sintomas mais comuns hemorragia gastrointestinal e dor abdominal. Outros sintomas incluem náuseas, vômitos, dor lombar, dor torácica e massa abdominal. 

 Como a apresentação clínica mais prevalente desta patologia é o sangramento gastrointestinal, os primeiros exames geralmente solicitados são a endoscopia e colonoscopia diagnósticas. O rompimento do PSAE pode ocorrer para o interior de um pseudocisto, para a cavidade peritoneal ou retroperitônio, sem hemorragia digestiva, ou pode fistulizar para a luz intestinal 20 , para a via biliar (hemobilia) ou para o interior do ducto pancreático 13 ^,^ 18 , condição denominada *hemosuccus pancreaticus* 16 ^,^ 21 ^-^ 23 . Neste caso, foi realizada EDA, tendo como indicação a hematêmese, sangramento direto para o trato gastrointestinal. O tratamento endoscópico não foi realizado devido à falta de evidência de sangramento ativo. 

 Para elucidação diagnóstica, prosseguiu-se com a RM, que evidenciou o volumoso PSAE. Nesse contexto, diversos exames de imagem fazem parte das opções diagnósticas, sendo a angiotomografia computadorizada (angioTC) e a RM os melhores exames não invasivos atualmente 8 ^,^ 19 . A RM possui a vantagem de não utilizar contraste iodado, oferecendo um estudo tão eficaz quanto a angioTC 8 , porém suas limitações incluem as contraindicações para pacientes portadores de marcapasso ou clipes metálicos de aneurismas, os que sofrem de claustrofobia e os que apresentam incapacidade de prender a respiração 19 . No estudo de tomografia computadorizada (TC) sem contraste, o PSAE pode ser identificado como uma região de intensificação focal cercada por fluido hipodenso. O PSAE apresenta atenuação aumentada quando sem contraste, mas sua porção perfundida é fortemente intensificada com contraste 19 . Com a TC com múltiplos detectores, a possibilidade de realizar reconstruções multiplanares com espessuras de corte submilimétricas, aliada à aquisição de imagens angiotomográficas, permite não apenas estabelecer o diagnóstico como também inferir qual o vaso acometido e definir a estratégia cirúrgica 8 ^,^ 21 . Neste caso, o diagnóstico inicial e o planejamento cirúrgico foram feitos com a RM, método diagnóstico disponível no momento da apresentação clínica. 

 O eco-Doppler colorido geralmente é a primeira modalidade de exame de imagem no diagnóstico de pseudoaneurismas periféricos 8 , visto que se trata de exame não invasivo que oferece o benefício de ser amplamente disponível, de baixo custo, com avaliação em tempo real e sem uso de contraste 4 . No entanto, esse exame é operador-dependente e pode ser limitado pela obesidade e sombra acústica, ocasionada pelo gás intestinal e pela aterosclerose 19 . Além disso, pequenas lesões podem passar despercebidas 4 ^,^ 19 . Neste caso relatado, foi indicada diretamente a RM devido à alta suspeição de PSAE e ao quadro emergencial, o que exigiu alta especificidade do método diagnóstico. 

 De posse da maior quantidade de informações possíveis a respeito da anatomia e do estadiamento da patologia, em conjunto com a análise das condições clínicas do paciente, é possível indicar com segurança o melhor tratamento. No passado, a ligadura da AE, com ou sem revascularização, e a esplenectomia associada ou não a pancreatectomia parcial eram os tratamentos usualmente utilizados 9 ^,^ 11 ^,^ 24 . O tratamento cirúrgico está associado a altas taxas de mortalidade (5-25% 10 ), além de haver casos com contraindicação relativa para laparotomia, como os associados a pancreatite aguda. A pancreatectomia distal associada ou não a esplenectomia apresenta taxa de mortalidade de 10-50% e sobrevida em 1 ano de 100% e em 5 anos de 85% 18 . Tessier et al. 4 advogam que o pseudoaneurisma associado à formação de pseudocisto pancreático é melhor tratado pela excisão cirúrgica devido à dificuldade de embolizar a grande cavidade de um pseudocisto. Nos pacientes que apresentam ruptura do pseudoaneurisma para cavidade livre do pseudocisto, a embolização pode ser adequada para resolver a instabilidade hemodinâmica, para posterior tratamento cirúrgico definitivo. As principais indicações de tratamento cirúrgico invasivo são a instabilidade do paciente para realização do procedimento angiográfico, a incapacidade de realizar a embolização e a persistência do sangramento ou ressangramento 6 . Neste caso em questão, a paciente apresentava anatomia favorável para o tratamento endovascular e era portadora de pancreatite crônica agudizada, quadro inflamatório que dificultaria a abordagem cirúrgica aberta. 

 Os novos métodos menos invasivos são preferidos, com melhores resultados e menor morbidade 9 ^,^ 10 ^,^ 11 ^,^ 25 ^,^ 26 . A técnica de embolização é associada a menor necessidade de transfusão e menor tempo de estadia no hospital, quando comparada à cirurgia aberta 24 ^,^ 27 . Em um estudo retrospectivo de pacientes portadores de aneurisma e pseudoaneurisma de artérias viscerais submetidos a tratamento cirúrgico no período entre janeiro de 2007 e abril de 2015 no departamento de cirurgia vascular da Cleveland Clinic, Batagini et al. 27 evidenciaram que o tratamento minimamente invasivo apresentou menor tempo operatório, menor perda sanguínea/taxa de transfusão e menor tempo de internação quando comparado ao tratamento cirúrgico convencional. Não encontraram diferenças nas taxas de complicações intra ou pós-operatórias, como isquemia de órgão, infarto agudo do miocárdio, insuficiência renal aguda, trombose venosa profunda e complicações respiratórias. Também não houve diferença significativa em relação ao sucesso técnico do procedimento. Durante o seguimento de médio prazo – média de 16 meses –, não houve diferença de sucesso clínico entre as duas abordagens. Na conclusão, os autores salientam a necessidade de estudos de acompanhamento de longo prazo para avaliar a durabilidade do tratamento endovascular, porém os resultados bem-sucedidos iniciais e as baixas taxas de reintervenção tornam a abordagem minimamente invasiva segura e factível. Há de se considerar que em casos de abdômen hostil, como desta paciente em questão, as vantagens da abordagem percutânea podem ser bem mais evidentes. 

 A arteriografia percutânea é considerada o método padrão-ouro e mais sensível para identificar aneurismas e pseudoaneurismas 6 . Provê uma avaliação vascular detalhada e está recomendada antes de qualquer procedimento cirúrgico eletivo, já que confirma o diagnóstico e determina a localização com sensibilidade de 94-100% 6 ^,^ 24 . A técnica de embolização é segura e pouco invasiva, mas apresenta taxa significativa de insucesso com 5% de reperfusão após o tratamento 7 . Spiliopoulos et al. 3 analisaram os resultados do tratamento endovascular de aneurismas e pseudoaneurismas viscerais em três departamentos europeus de radiologia intervencionista entre 2000 e 2010. No total, foram 21 aneurismas e 37 pseudoaneurismas viscerais tratados. Relataram sucesso técnico em 100% dos casos, com taxa de reintervenção de 6,1% no grupo de pseudoaneurismas e de 14,2% no grupo de aneurismas. A taxa de mortalidade geral do procedimento foi de 3% no grupo dos pseudoaneurismas e de 0% no grupo dos aneurismas. Eles concluíram que o tratamento endovascular é seguro e efetivo, com baixa morbidade e mortalidade. 

 O tratamento endovascular pode ser feito por diversas técnicas com o uso de molas, N-butil cianoacrilato (NBCA), copolímero de etileno e álcool vinílico (Onyx®), trombina e stent revestido 8 . Como o pseudoaneurisma apresenta perda da integridade da parede vascular, a embolização propriamente dita não deve ser a terapia isolada de escolha, visto que o conteúdo emboligênico ficará contido por uma parede virtual, incorrendo sério risco de nova ruptura. Há relato na literatura de migração de um stent revestido para o interior do estômago após o tratamento de pseudoaneurisma esplênico 28 . O procedimento percutâneo para embolização consiste na cateterização superseletiva da artéria envolvida e a embolização distal e proximal a lesão e do saco endoluminal do pseudoaneurisma, com uso de molas ou de NBCA 6 . É essencial excluir ambos os vasos aferente e eferente para reduzir o risco de reperfusão anterógrada e retrógrada 3 ^,^ 7 ^,^ 26 . Com a necessidade de posicionamento das molas no ramo eferente e então no ramo aferente para se obter exclusão completa, a perfusão do órgão será ao menos parcialmente mantida por vasos colaterais 26 . 

 O uso de molas e micromolas, que são os agentes de escolha no tratamento de aneurismas e pseudoaneurismas 3 ^,^ 25 ^,^ 29 , pode resultar em embolização incompleta ou ineficaz devido às seguintes causas: (1) artérias nutridoras do pseudoaneurisma finas ou tortuosas; (2) rede de colaterais para o pseudoaneurisma; (3) compactação inadequada do “ninho” de molas; ou (4) deficiência de coagulação 3 ^,^ 25 . Duas técnicas endovasculares com uso de molas são preconizadas. A técnica de compactação do ninho (*coil packing*) é usada para embolizar o saco do aneurisma em si. A técnica de exclusão (*coil trapping* ) consiste na oclusão das artérias proximais e distais para prevenir o reenchimento da lesão 7 ^,^ 8 . 

 Kingma et al. 7 citam um caso em que foi utilizada a técnica de exclusão; porém, devido ao vasoespasmo intenso consequente à progressão da ponta do cateter, não foi possível a embolização da artéria aferente. Tal fato impossibilitou também o implante de stent revestido, sendo necessário tratamento cirúrgico aberto com esplenectomia e pancreatectomia caudal. Os autores advogam que o uso de microcateter poderia ter permitido a cateterização distal sem vasoespasmo ou dissecção da artéria. No presente caso, utilizamos a técnica coaxial para posicionamento do sistema de trabalho no segmento proximal da AE e a técnica de microcateterismo com microcateter e microfio-guia para embolização da artéria aferente e eferente ao pseudoaneurisma ( Figura 5 ). 

 O NBCA é um líquido claro à temperatura ambiente que rapidamente se solidifica ao contato com fluidos iônicos, como solução salina e sangue 29 . Como agente emboligênico, o NBCA possui duas vantagens em relação às molas: oclusão imediata da artéria embolizada e possibilidade de ser implantado mais distalmente que as micromolas 9 . Outros benefícios dessa cola incluem: baixa viscosidade, que permite embolização distal quando a cateterização é impossível devido à dificuldade anatômica; embolização das artérias colaterais; e a não dependência da atividade de coagulação 25 . Entretanto, o NBCA pode polimerizar precocemente e ocasionar a aderência do cateter no vaso. Pode ocorrer também a polimerização atrasada, que resulta em embolia distal irreversível 30 . Portanto, para o uso efetivo do NBCA, é necessário o controle sobre o volume injetado e a velocidade de infusão 9 , o que dificulta a execução para os não familiarizados com o método. No presente caso, com alto fluxo arterial em vaso de médio calibre, o uso de cola líquida de alta polimerização seria contraindicado pelo alto risco de embolia distal e consequente isquemia esplênica. 

 O Onyx® é outra alternativa à embolização por mola e pode ser utilizado atualmente de forma semelhante ao NBCA. Há relatos na literatura da indicação na neurointervenção para embolização direta do saco aneurismático. No entanto, a fórmula de alta densidade (500) utilizada para este propósito foi retirada de comercialização. As apresentações disponíveis no mercado brasileiro são de densidades menores para uso periférico. O Onyx® é um agente líquido embólico não adesivo dissolvido no dimetilsulfóxido (DMSO) 30 . Quando o DMSO se difunde para fora da mistura, o Onyx® se torna um sólido esponjoso elástico, ocasionando a exclusão do saco ou do vaso ao preenchê-lo como um polímero elástico que se acomoda à parede, ocupando todo o lúmen. Não há risco de adesão permanente do cateter à parede, risco inerente ao uso do NBCA. O Onyx® vem sendo usado com sucesso em aneurismas que não possuem indicação para uso de molas ou na falha da embolização por outros materiais. No entanto, atualmente há poucos dados de longo prazo sobre a indicação em aneurismas viscerais verdadeiros ou não verdadeiros 30 . 

 Na literatura, a taxa de sucesso da embolização varia de 75-100% com morbidade estimada em 14-25% 26 . Ballinas-Oseguera et al. 6 citam que, nos casos de embolização bem-sucedida, o controle da hemorragia ocorre em 93% dos casos na primeira arteriografia e em 100% após a segunda sessão. Tais dados se aproximam do estudo de Laganà et al. 26 , que trataram 25 de 29 pacientes portadores de aneurismas de artérias viscerais por embolização e obtiveram exclusão imediata dos aneurismas em todos os casos. Dos quatro pacientes restantes, dois foram tratados com stent revestido e outros dois por injeção de trombina e embolização com molas na artéria aferente. A taxa de complicação foi de 27,6% (sete casos de isquemia do baço e uma oclusão de stent revestido), e em 10,3% dos casos houve reperfusão no primeiro mês de acompanhamento, todos tratados com êxito por técnicas endovasculares. A taxa de sucesso primário foi de 89,7% e de sucesso secundário, 100%. A taxa de reperfusão foi de 5%, e todos os casos ocorreram logo após o tratamento. Nenhuma recanalização foi reportada em médio e longo prazo. 

 Entre as desvantagens da embolização do PSAE, pode-se citar uma taxa de infarto esplênico de 40%. Grandes infartos esplênicos são mais comuns com embolização do terço distal da artéria esplênica ou no hilo. Tais pacientes podem apresentar quadro de dor abdominal intensa com necessidade de internação hospitalar para controle álgico. Pode haver necessidade de esplenectomia ou drenagem de abscesso esplênico 4 . Neste relato de caso, o pseudoaneurisma estava localizado no ramo inferior da AE, evoluindo sem sinais de infarto do órgão por provável perfusão preservada pelo ramo superior pérvio. Há relatos de migração de materiais utilizados na embolização para artérias viscerais, aorta e trato gastrointestinal, e a exclusão da circulação distal pode comprometer a função do órgão 10 . A migração do conteúdo emboligênico extra-arterial ocorre se a embolização do saco aneurismático for realizada, visto que no pseudoaneurisma não há parede arterial. Portanto, preconizamos a utilização da técnica de exclusão arterial aferente e eferente (*coil trapping* ) como descrito acima. 

 A exclusão do aneurisma com stent revestido oferece alternativas às desvantagens da embolização, pois permite hemostasia e trombose efetiva do pseudoaneurisma sem risco de ressangramento, com manutenção do lúmen e fluxo vascular para o órgão 2 ^,^ 26 ^,^ 30 . Está bem indicada nos casos de pseudoaneurisma de colo largo 8 . Essa técnica oferece menor morbidade quando comparada à cirurgia aberta e à embolização. No entanto, fica restrita a uma anatomia adequada. Entre as condições anatômicas favoráveis estão o colo distal e proximal mínimos (5-15 mm de comprimento), calibre adequado 2 , localização proximal da lesão na AE e ausência de ramificação arterial 10 . Devido à intensa tortuosidade da artéria esplênica, alta incidência desse tipo de lesão próxima ao hilo e rigidez dos dispositivos de entrega, frequentemente as condições anatômicas não são ideais 3 ^,^ 26 ^,^ 30 . O tratamento com stent revestido é usualmente difícil devido aos pequenos diâmetros das artérias viscerais. Entre as causas de insucesso reportadas estão a recanalização por colaterais e remodelagem vascular 26 . Pode ocorrer também hiperplasia intimal nas bordas do stent recoberto 30 . A presença de sepse não é uma contraindicação absoluta ao seu uso, mas exige vigilância durante o acompanhamento pós-operatório 8 ^,^ 10 . Reed et al. realizaram estudo retrospectivo dos pacientes portadores de aneurisma de artéria esplênica tratados entre 2009 e 2014 com stent revestido ou embolização de resgate na falha do primeiro método. Foram 10 pacientes, com diâmetro médio do aneurisma de 2,8 ± 1,3 cm e tamanho médio do stent revestido de 6x100 mm, média de 1,5 stent implantado (variando de um a quatro). A taxa de sucesso técnico foi de 80%, sendo as duas falhas técnicas relativas à intensa tortuosidade do vaso. Nos dois casos, o tratamento foi efetivado com uso de molas ou *plug* oclusor Amplatzer. No presente procedimento, devido à intensa tortuosidade da AE, optamos pela técnica de embolização com micromolas, associando a técnica coaxial para aumentar o sustento dos sistemas de trabalho. 

 Shrivastava et al. 31 relatam um caso tratado por punção percutânea direta do pseudoaneurisma, guiada por ultrassonografia. A intensa tortuosidade da AE impossibilitou o cateterismo seletivo. A embolização foi realizada com uso de molas, resultando em sucesso técnico e manutenção da perviedade da AE. Há, também, relatos de injeção de trombina guiada por ultrassonografia em pseudoaneurismas viscerais, com sucesso 8 . 

 Independentemente da técnica utilizada, grande parte das complicações reportadas na literatura compreendem isquemia da parte superior do trato gastrointestinal, em 30-66% dos casos. Podem ocorrer infartos esplênicos, que se resolvem espontaneamente ou necessitam de esplenectomia 2 ^,^ 18 ; complicações técnicas como migração do material embolizante ou stent revestido 28 ; ou complicações do acesso cirúrgico/punção. Os pacientes podem apresentar dor, febre e aumento transitório de enzimas pancreáticas ou hepáticas (síndrome pós-embolização) 26 . No presente caso, a paciente evoluiu com estabilidade hemodinâmica, sem complicações relativas à técnica de embolização, exceto por dor discreta em hipocôndrio esquerdo no primeiro dia pós-operatório. 

## CONCLUSÃO

 O tratamento minimamente invasivo é relatado como eficaz para o tratamento de PSAE, assim como outros pseudoaneurismas viscerais, inclusive no contexto de instabilidade hemodinâmica 3 . A escolha da técnica deve se basear nas condições clínicas e anatômicas do paciente e na habilidade e familiarização do cirurgião ou radiologista intervencionista com o método. Cabe ressaltar a importância do tratamento não só do pseudoaneurisma em si, mas também dos vasos aferentes e eferentes, para diminuir a chance de recorrência.
